# Detection of genuine grass pollen sensitization in children by skin testing with a recombinant grass pollen hybrid

**DOI:** 10.1111/pai.12991

**Published:** 2018-11-25

**Authors:** Nikolaos Douladiris, Victoria Garib, Margit Focke‐Tejkl, Rudolf Valenta, Nikolaos G. Papadopoulos, Birgit Linhart

**Affiliations:** ^1^ Allergy Department, 2nd Pediatric Clinic University of Athens Athens Greece; ^2^ Division of Immunopathology, Department of Pathophysiology and Allergy Research, Center of Pathophysiology, Infectiology and Immunology Medical University of Vienna Vienna Austria; ^3^ NRC Institute of Immunology FMBA of Russia Moscow Russia; ^4^ Laboratory for Immunopathology, Department of Clinical Immunology and Allergy Sechenov First Moscow State Medical University Moscow Russia; ^5^ Division of Infection, Immunity & Respiratory Medicine University of Manchester Manchester UK

**Keywords:** allergen, allergy, molecular diagnosis, pollen, recombinant hybrid allergen, respiratory allergy, skin prick test

## Abstract

**Background:**

Skin testing represents a commonly used first diagnostic method in clinical practice, but allergen extracts may vary in composition and often contain cross‐reactive allergens and therefore do not always allow the precise identification of the sensitizing allergen source. Our aim was to investigate the suitability of a single recombinant hybrid molecule, consisting of the four major timothy grass pollen allergens (Phl p 1, Phl p 2, Phl p 5, and Phl p 6) for in vivo diagnosis of genuine grass pollen allergy in children suffering from pollinosis.

**Methods:**

Sixty‐four children aged from 6 to 17 years with a positive skin reaction and/or specific IgE to grass pollen extract and respiratory symptoms of pollinosis as well as 9 control children with allergy to other allergen sources were studied. SPT was performed with the recombinant hybrid, the four recombinant timothy grass pollen allergens, and grass pollen extract. Specific IgE reactivity to 176 micro‐arrayed allergen molecules was determined using ImmunoCAP ISAC technology. IgE reactivity to the hybrid was detected by non‐denaturing RAST‐based dot blot assay.

**Results:**

Genuine grass pollen sensitization was confirmed in 94% of the children with positive SPT to grass pollen extract by SPT and IgE reactivity to the hybrid. The four hybrid‐negative children showed IgE reactivity to cross‐reactive allergens such as Phl p 4, Phl p 11, and Phl p 12 and had also sensitizations to pollen allergens from unrelated plants.

**Conclusions:**

The recombinant hybrid molecule represents a useful tool for in vivo diagnosis of genuine grass pollen sensitization.

## 1 INTRODUCTION

Grass pollen allergy is an increasing health problem in Western countries limiting patients’ quality of life but also representing a relevant socioeconomic burden.^1^ A European skin test study revealed an average sensitization rate to grass pollen, which is clinically relevant, of 33%.^2^ Sensitization to grass pollen allergens often starts early in childhood with IgE reactivity to the major allergen Phl p 1 followed by IgE recognition of additional allergens.^3^ Among the numerous grass pollen allergens, the group 1, 2, 5, and 6 allergens are recognized most frequently by IgE antibodies from grass pollen allergic patients and elicit most of the allergic symptoms (ie, rhinitis, conjunctivitis, asthma).^4^, ^5^, ^6^ Specific immunotherapy, the only causative and disease‐modifying treatment, leads to a long‐lasting clinical benefit, and it was shown in children with seasonal rhinoconjunctivitis that early AIT prevents the progression to severe and chronic forms of the disease such as asthma.^7^, ^8^ However, the accurate prescription of SIT depends on the correct identification of the culprit allergens, which can be facilitated by molecular allergy diagnosis.^9^, ^10^, ^11^ Skin testing represents a commonly used first diagnostic method in clinical practice, but its informative value is limited by the use of allergen extracts with varying and sometimes undefined composition.^12^, ^13^ In addition, the presence of cross‐reactive allergens in various extracts often impedes the determination of the sensitizing allergen source, especially in polysensitized patients.^9^ In southern Europe, the overlapping pollination seasons and an abundance of multisensitization profiles make it even more difficult to distinguish among sensitizations to different pollen allergens.^14^ Moreover, the number of commercially available skin test solutions rapidly decreases due to EU directives making marketing authorization more difficult in the future.^15^

In vitro diagnostic tests based on single allergen molecules were recognized as a helpful tool, but they require access to laboratory testing facilities and blood sampling.^14^, ^16^, ^17^, ^18^, ^19^ Component‐based in vivo tests are currently not available, though several studies have already made use of recombinant allergens for provocation testing. 20 A recombinant hybrid molecule which was constructed out of the four major allergens from timothy grass (ie, Phl p 1, Phl p 2, Phl p 5, Phl p 6) was shown to contain most of the grass pollen‐specific IgE epitopes.^21^ It has already been successfully used for in vivo diagnosis of grass pollen allergy in a SPT study in a French population of adult grass pollen allergic patients.^22^ Other grass pollen allergens were not included, due to their cross‐reactivity with homologous allergens from other allergen sources, such as Phl p 7 and Phl p 12, and their minor clinical relevance as poor elicitors of allergic reactions, such as Phl p 4 and Phl p 13.^6^, ^9^ Due to extensive cross‐reactivity, Phl p 3 could be substituted by the presence of Phl p 2.^23^ Here, we investigated the suitability and safety of this hybrid molecule and the single recombinant grass pollen allergens Phl p 1, Phl p 2, Phl p 5, and Phl p 6, compared to natural grass pollen extracts, for in vivo diagnosis of genuine grass pollen sensitization in children with pollinosis.

## 2 METHODS

### 2.1 Recombinant timothy grass pollen allergens, hybrid, allergen extracts

Recombinant grass pollen allergens Phl p 1, Phl p 2, Phl p 5, and Phl p 6 were obtained from Biomay AG (Vienna, Austria). The recombinant hybrid molecule consisting of Phl p 1, Phl p 2, Phl p 5, and Phl p 6 was expressed in *Escherichia coli* and purified as described.^21^ Protein concentrations were measured by Micro BCA (Pierce, Rockford, IL, USA), and purity of the recombinant proteins was analyzed by SDS‐PAGE using ImageJ software (National Institutes of Health, open source). Proteins were dissolved in distilled water, filter‐sterilized (0.2 µm), and applied to ethidium bromide plates with λ DNA as a standard to confirm the absence of host DNA. Timothy grass pollen extract and grass mix (including cocksfoot, sweet vernal grass, rye grass, meadow grass, timothy grass pollen extracts) were purchased from Stallergenes (Antony, Hauts‐de‐Seine, France).

### 2.2 Pollen allergic children

Sixty‐four allergic children between 6 and 17 years of age with symptoms of pollinosis were studied after permission from the Institutional Ethics Committee and informed consent from the parents. They had a positive SPT to grass pollen extracts (grass mix, timothy grass) and/or grass pollen allergen‐specific IgE measured by ImmunoCAP (gx2: bermuda grass, timothy grass, rye grass, Kentucky blue grass, Johnson grass, bahia grass; g6: timothy grass) (Thermo Fisher, Uppsala, Sweden). The demographic, clinical, and serologic characteristics of the pollen allergic children are summarized in Table S1. Serum samples were analyzed anonymously with permission from the ethics committee of the Medical University of Vienna, Austria (EK565/2007).

### 2.3 ImmunoCAP, ISAC chip technology, IgE dot blot

Quantitative measurement of specific IgE for grass pollen allergens and total IgE was performed by ImmunoCAP (gx2, g2, g6) (Thermo Fisher). IgE values were expressed as kU_A_/L for allergen‐specific IgE and kU/L for total IgE. The sensitization profile of grass pollen allergic patients was determined by ImmunoCAP ISAC technology, using a microarray containing 176 purified allergens as previously described.^16^ IgE values were expressed as standardized units (ISU) by interpolating the mean fluorescence value with a previously established reference curve. Results ≥0.3 ISU were considered as positive.

For dot blot analysis, 1 µg of the hybrid was dotted on nitrocellulose membranes (Schleicher & Schuell, Dassel, Germany) and exposed to 1:5 diluted sera from children with negative SPT to the hybrid. Sera from an adult grass pollen allergic patient (positive control), a non‐allergic individual, and buffer (negative controls) were included. Bound IgE antibodies were detected with 1/15 diluted ^125^I‐labeled rabbit anti‐human IgE antibodies (IBL, Hamburg, Germany) and visualized by autoradiography.

### 2.4 Skin prick testing

SPTs were performed at the Allergy Department, 2nd Pediatric Clinic, University of Athens, “P&A Kyriakou” Children's Hospital, according to the guidelines of the European Academy of Allergy and Clinical Immunology.^24^ SPT was done by a single qualified physician, with approval of the Institutional Ethics Committee, after written informed consent was obtained from the parents and oral consent from the patients. SPT was performed on the forearms using histamine hydrochloride (positive control), physiologic saline (negative control), grass pollen allergen extracts (timothy grass, grass mix), and purified recombinant Phl p 1, Phl p 2, Phl p 5, Phl p 6, and the recombinant hybrid (30 µg/mL). Results were read after 20 minutes. Mean wheal diameters >3 mm were considered positive. All children were followed on site for 2 hours and instructed to report any reaction or adverse event within 72 hours.

### 2.5 Statistical analysis

Mean and median values were calculated using GraphPad Prism software 5.0 (GraphPad Software, San Diego, CA, USA).

## 3 RESULTS

### 3.1 Expression and purification of a recombinant hybrid allergen containing Phl p 1, Phl p 2, Phl p 5, and Phl p 6

**Figure 1:**
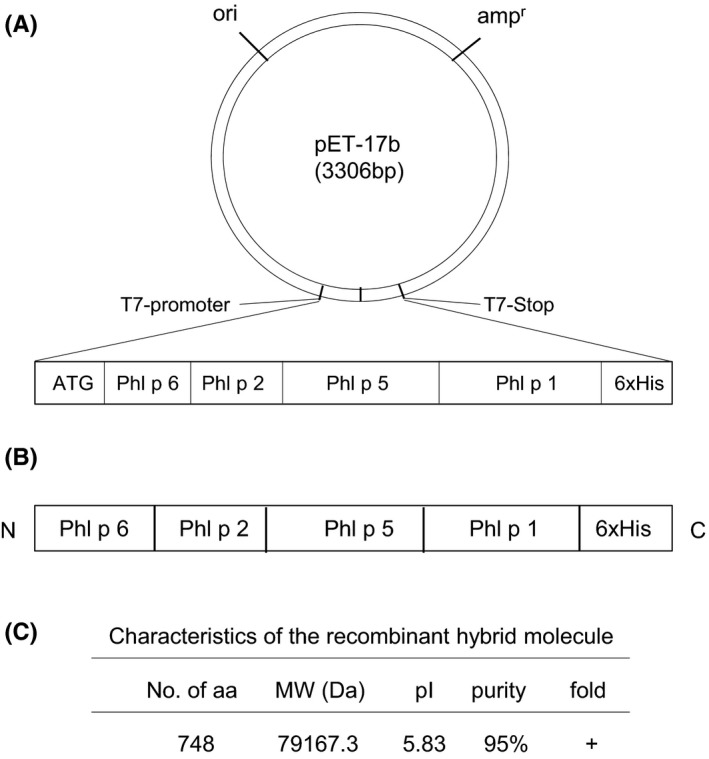
Representation and characteristics of the hybrid molecule. A, Construction of an expression plasmid (pET‐17b) containing the cDNA coding for the hybrid. The hybrid‐encoding cDNA was inserted into the multiple cloning site of plasmid pET17b, and a start codon and a 6xhistidine tag (6xHis) were introduced at the 5’ and 3’ end of the hybrid‐encoding sequence, respectively. B, Schematic representation and C, characteristics of the hybrid protein

A recombinant hybrid molecule consisting of the major timothy grass pollen allergens Phl p 1, Phl p 2, Phl p 5, and Phl p 6 was generated by fusing the cDNA sequences of the single allergens and cloning the resulting gene into the expression vector pET‐17b in the order Phl p 6‐Phl p 2‐Phl p 5‐Phl p 1 followed by a 3’ 6xhistidine tag (Figure 1A). The hybrid was expressed in *E coli* transformed with the plasmid yielding high amounts of the fusion protein (Figure 1B). Purification was performed by affinity chromatography via the 6xhistidine tag with a purity of >95%.^21^ Circular dichroism analysis revealed a secondary structure of the hybrid comparable to a mixture of the single grass pollen allergens (data not shown). Main characteristics of the recombinant hybrid molecule are summarized in Figure 1C.

### 3.2 Diagnosis of genuine grass pollen allergy using the recombinant grass pollen allergens Phl p 1, Phl p 2, Phl p 5, and Phl p 6, and the hybrid

Sixty‐four children aged from 6 to 17 years were subjected to SPT with Phl p 1, Phl p 2, Phl p 5, Phl p 6, and the hybrid molecule. All patients had a positive skin reaction and/or specific IgE to grass pollen extract and symptoms of pollinosis during grass pollen season (rhinitis: 95.3%; conjunctivitis: 62.5%; asthma: 53.1%). A detailed description of the demographic, clinical, and serologic characteristics is listed in Table S1. In addition, nine children without symptoms of grass pollen allergy but with allergy to other allergen sources were included in our investigation as negative controls (Table S2).

**Figure 2:**
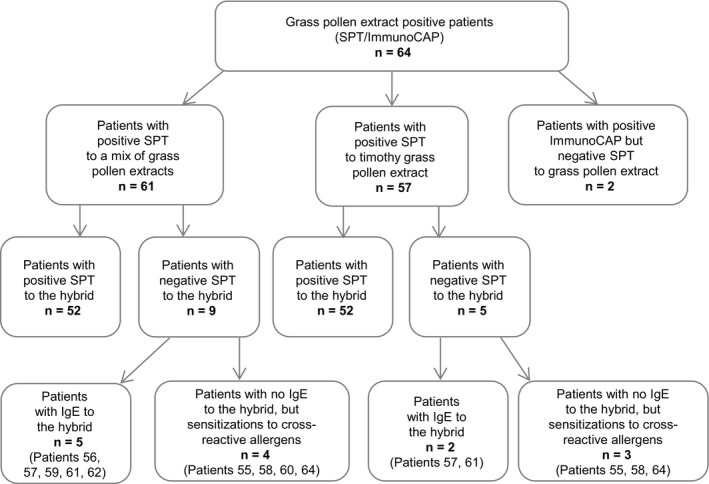
Flow diagram for the diagnosis of genuine grass pollen allergy based on the hybrid molecule

Sixty‐one of the 64 children developed a positive skin reaction to grass mix (mean wheal diameter 6.2 mm ±2.6) and 57 of 64 children had a positive SPT to timothy grass pollen extract (mean wheal diameter 5.2 mm ±2.2) (Figure 2, Table S3). Two patients had no positive SPT to any grass pollen extract (patients 54 and 63) and were therefore excluded from further analysis. Within the control group, no positive skin reaction to grass pollen extracts or an IgE response to timothy grass pollen allergens was detected (Table S2).

All patients and controls were tested for skin reactivity to the hybrid molecule and the single recombinant grass pollen allergens (Table 1, Table S3). None of the children reported any large local, systemic, late phase, or delayed‐type reaction or any adverse event related to skin prick testing with purified recombinant Phl p 1, Phl p 2, Phl p 5, Phl p 6, and the hybrid molecule. A positive skin reaction to the hybrid molecule was observed in 53 of the 62 children with a positive SPT to grass pollen extract (85%, mean wheal diameter 4.4 mm ±1.8). Positive SPT responses to the single grass pollen allergens were detected as follows: Phl p 1:68%, mean wheal diameter 3.9 mm ±0.8; Phl p 2:34%, mean wheal diameter 6.9 mm ±2.8; Phl p 5:64%, mean wheal diameter 6.6 mm ±2.3; Phl p 6:40%, mean wheal diameter 7.0 mm ±3.1 (Table 1). All 53 patients with a positive skin reaction to the hybrid also had a positive reaction to at least one single grass pollen allergen (Table S3). It is of note that among the patients with a positive SPT to the grass mix (n = 61), nine patients were hybrid‐negative, while among the children with a positive SPT result to timothy grass pollen extract (n = 57), only five were negative for the hybrid (Figure 2, Table S3). We further analyzed allergen‐specific sIgE levels of these 9 patients (patients 55‐62, 64) by allergen‐chip measurement and the hybrid‐specific IgE response by dot blot (Table 2, Figures S1 and S2). We found that 5 of 9 children had specific IgE to the hybrid (patients 56, 57, 59, 61, 62). Patients 55, 58, 60, and 64 showed a negative SPT and dot blot result to the hybrid, and a detailed description of these patients is given in Table 2. Patient 55 suffered from mild, obstructive rhinitis with exacerbations from February to May and September to December, conjunctivitis, asthma, and food‐ and exercise‐induced anaphylaxis. Measurement of sIgE revealed a pronounced sensitization to LTPs, but also to the timothy grass pollen allergens Phl p 11 and Phl p 4, which could explain the positive SPT result to grass pollen extract. A sensitization to Phl p 1 could be detected, though at a low level (0.51 ISU), which might have not translated into clinical symptoms yet, as the SPT to Phl p 1 and the hybrid were both negative in this patient. In patients 58 and 59, who were diagnosed with moderate (patient 58), and mild (patient 60) rhinitis and persistent, post‐viral asthma, an Ole e 1‐specific IgE response (patient 58:46.68 ISU, patient 60:0.42 ISU) was detected. IgE to Phl p 4 was measured in these patients, while allergic symptoms were most likely elicited due to the sensitization to olive pollen. Patient 64, who developed conjunctivitis and moderate rhinitis from March to June, had IgE to profilin including Phl p 12, which could be responsible for the positive SPT to grass pollen extract, while clinical symptoms could be attributed to the sensitization to weed pollen allergens. Low levels of Phl p 1‐specific IgE (0.32 ISU) but a negative SPT result to Phl p 1, as observed for patient 55, were also detected in this patient (Table 2).

**Table 1:** Skin reactivity of grass pollen allergic children (n = 64)

	Grass mix	Timothy grass	Phl p 1	Phl p 2	Phl p 5	Phl p 6	hybrid
Number of SPT‐positive patients	61	57	42	21	40	25	53
Mean wheal diameter (mm)	6.32	5.22	3.92	6.86	6.6	7.02	4.42
min‐max	3‐17	3‐11.5	3‐6	4‐15	3.5‐13	3‐13	3‐12
Median	6	4.5	4	6.5	6.25	7	4

**Table 2:** Characteristics of patients with positive SPT to grass pollen extract but negative SPT and dot blot result to the hybrid

Patients	55	58	60	64
Age	15	8	6	14
Family history	No	Yes	Yes	Yes
Asthma	No	Yes	Yes	No
Severity	Na	Mild persistent	Moderate persistent	Na
Phenotype	Na	Post‐viral	Post‐viral	Na
Rhinitis	Yes	Yes	Yes	Yes
Severity	Mild	Moderate	Mild	Moderate
Phenotype	Obstructive	Sneezing	Obstructive	Obstructive
Months of exacerbation	II‐V; IX‐XII	nk	I‐V; IX‐XII	III‐VI
Conjunctivitis	Yes	No	No	Yes
Atopic dermatitis	No	Yes	Yes	No
Food allergy	Food and exercise‐induced anaphylaxis	No	No	No
Total IgE (kU/L)	249	360	105	439
ISU‐IgE positive	Ole e 7; LTP 0.74	Ole e 1; 46.68	Ole e 1; 0.42	Amb a 1; pectate lyase 0.99
	Pla a 3; LTP 3.46			Art v 1; defensin 19.78
	Ara h 9; LTP 0.33			Bet v 2; profilin 16.26
	Cor a 8; LTP 1.66			Hev b 8; profilin 23.13
	Jug r 3; LTP 1.39			Mer a 1; profilin 27.21
	Pru p 3; LTP 6.67	Pru p 3; LTP 0.6		Pru du 4; profilin 7.5
	Pru du 3; LTP 0.55			Profilin; 7.83
				Par j 2; LTP 51.33
Explanation of positive SPT to grass pollen				
Phl p 1	0.51			0.32
Phl p 11	0.44			
Phl p 4	3.51	0.59	1.52	
Phl p 12				0.72
Conclusion	Sensitization to olive and plane LTP syndrome	Sensitization to olive pollen	Sensitization to olive pollen	Sensitization to weed pollen

nk, not known; na, not applicable.

In addition, we found that sIgE measurements and SPT results to the single grass pollen allergens Phl p 1, Phl p 2, Phl p 5, and Phl p 6 were in good agreement (Table S4). IgE reactivity and SPT results matched in >95% of the patients for Phl p 2 and Phl p 5, and in 89% of the patients for Phl p 6. Regarding Phl p 1, the chip measurement was more sensitive than skin testing, as Phl p 1‐specific skin reactions and IgE reactivity were in agreement in 73% of the patients. In 16 patients, a sensitization to Phl p 1 was detected, which did not translate into a mast cell degranulation in the skin (Table S5).

In summary, diagnosis based on the hybrid molecule identified 57 of 61 (93.4%) patients with positive SPT to a grass mix and 54 of 57 (94.7%) patients with positive SPT to timothy grass pollen extract as genuine grass pollen‐sensitized (Figure 2).

## 4 DISCUSSION

We generated a recombinant hybrid molecule consisting of the four major timothy grass pollen allergens, Phl p 1, Phl p 2, Phl p 5, and Phl p 6. As the hybrid solely contains grass pollen‐specific epitopes, which are restricted to grass pollen and do not occur in other allergen sources, IgE recognition of the hybrid indicates a specific sensitization to grass pollen. The hybrid was already successfully used for in vitro serologic testing and in vivo diagnosis of grass pollen allergy in adult patients, demonstrating that all grass pollen allergic patients showed IgE reactivity as well as a positive skin reaction to the hybrid.^21^, ^22^ In this work, we investigated its suitability and safety for in vivo diagnosis of genuine grass pollen allergy in 64 children from southern Europe suffering from pollinosis. In patients from this area, the determination of the sensitizing allergen source is challenging due to overlapping periods of symptoms and flowering of allergenic plants and an abundance of multisensitization profiles. Distinguishing sensitization to grass, olive, and weed pollen is particularly difficult due to the high prevalence of these allergen sources and the presence of cross‐reactive allergens in pollen.^14^ In particular, for children the early and correct immunotherapy prescription is of great importance, as it has been shown that SIT does not only have an immediate clinical benefit, but also prevents the progression of allergic rhinitis to asthma.^8^

All investigated children had IgE and/or a positive skin reaction to grass pollen extract, but also multiple sensitizations to other pollen‐derived allergens were detected using an allergen microarray; notably, about 42% of the children had IgE to Ole e 1, which is a diagnostic marker for sensitization to olive pollen.^25^ We found that 93% of patients with a positive skin test to a mix of grass pollen extracts and 95% of patients with a positive skin test to timothy grass pollen extract also had a positive reaction to the hybrid, either by skin testing or serology. Those children were identified as genuinely grass pollen‐sensitized, and specific immunotherapy with grass pollen extract would represent an appropriate treatment. In contrast, four children did not react to the hybrid. They had sensitizations to other cross‐reactive grass pollen allergens such as Phl p 11, Phl p 12, and Phl p 4, which might explain the positive skin prick test results to grass pollen extract. Two of the hybrid‐negative children also had very low levels of Phl p 1‐specific IgE. However, allergic symptoms did not occur during the main flowering period of grasses in these children, indicating that the sensitization had not reached clinical relevance yet. Instead, allergic symptoms could be explained by sensitization to olive, plane, and weed pollen in the hybrid‐negative patients, and AIT against those allergen sources would therefore be an appropriate treatment. Moreover, for two of those children asthmatic reactions were attributed to viral infections. SIT with grass pollen extract would not have been useful for the hybrid‐negative patients. Thus, diagnosis based on the hybrid molecule allowed identifying genuine grass pollen allergy in the polysensitized children. We suggest that the sensitivity of in vivo diagnosis with the hybrid might be even increased by using a higher concentration comparable to a previous study, as no side effects during skin testing were observed in any of the investigated children.^22^

The combination of the 4 major grass pollen allergens was equally suited for the in vivo detection of grass pollen allergy by skin testing as the hybrid. However, considering the high costs for manufacturing and market authorization, the construction of a single hybrid molecule consisting of the major allergens from a complex allergen source may represent a big advantage.

Furthermore, we suggest that a vaccine comprising Phl p 1, Phl p 2, Phl p 5, and Phl p 6 will be suitable for the vast majority of grass pollen allergic patients, as they are only present in grass pollen and represent strong elicitors of allergic reactions.^6^ Phl p 7 and Phl p 12 are recognized only by few grass pollen allergic patients and therefore do not represent essential ingredients of a grass pollen vaccine. Such a vaccine could be based on the hybrid protein described by us, a mix of recombinant Phl p 1, Phl p 2, Phl p 5, and Phl p 6, or a mix of hypoallergenic derivatives of the four allergens such as the new recombinant grass pollen vaccine BM32.^26^, ^27^, ^28^, ^29^, ^30^

In clinical practice, skin testing still represents the first‐line diagnostic method, as it is fast, relatively cost‐effective, and has a low complication rate. Notably, about 50% of approved extract‐based test solutions disappeared from the market in the recent years due to the introduction of a more rigorous EU legislation.^15^ Considering that for all important allergen sources, the marker allergens indicating a specific sensitization are known, recombinant allergens and allergen derivatives as the recombinant grass pollen hybrid could replace traditional allergen extracts for a more precise in vivo provocation testing in the future.

## CONFLICT OF INTEREST

Rudolf Valenta has received research grants from Biomay, Vienna, Austria, and Viravaxx, Vienna, Austria, and serves as a consultant for both companies.

## AUTHORS’ CONTRIBUTIONS

N.D, VG, and BL contributed to acquisition of data. NGP, VR, and B.L were involved in the conception and design of the study. ND, VG, MFT, RV, NGP, and BL analyzed and interpreted the data. BL and RV wrote the original draft. ND, VG, MFT, RV, NGP, and BL reviewed and edited the manuscript.
