# Supplementary material for: Detection of genuine grass pollen sensitization in children by skin testing with a recombinant grass pollen hybrid
Source: Pediatr Allergy Immunol. 2018 Nov 25;30(1):59–65. doi: 10.1111/pai.12991 (PMC6378406; doi:10.1111/pai.12991)
Supplement: Supplementary file 1 [file PAI-30-59-s001.pdf]

Supplementary Table S1. Demographic, clinical, and serologic characteristics of grass pollen allergic children (n=64).

patient	sex	age	family history	asthma	severity	phenotype	rhinitis	severity	persistence	phenotype	conjunctivitis	AD	Food Allergy	total IgE kU/L	g6 kUA/L
1	f	11	y	y	1	2	y	2	1	1	y	y	n	143	7.38
2	m	6	y	y	1	2	y	2	2	1	y	n	n	56.6	56.5
3	m	12	y	y	1	1	y	1	1	1	y	n	n	1756	77.9
4	m	7	y	y	1	1	y	nk	nk	nk	y	n	n	91.4	nd
5	m	12	y	n	0	0	y	2	1	1	y	n	n	502	nd
6	f	11	y	n	0	0	y	1	1	1	y	y	y	180	3.97
7	m	15	y	y	1	1	y	2	1	1	y	n	n	1063	nd
8	f	9	y	y	2	2	y	2	2	1	y	y	y	nd	nd
9	m	12	y	y	1	3	y	1	1	2	nk	n	nk	486	nd
10	m	10	y	y	2	2	y	2	2	2	y	n	n	nd	nd
11	f	14	n	n	0	0	y	2	2	1	n	n	nk	51.9	nd
12	m	15	n	y	2	2	n	0	0	0	n	y	n	nd	nd
13	m	10	n	y	2	2	y	2	2	1	y	y	n	290	100
14	m	11	y	n	0	0	y	2	2	2	y	n	n	314	nd
15	m	12	y	n	0	0	y	2	2	1	y	y	y	2000	>100
16	m	15	y	n	0	0	y	2	2	2	y	n	n	317	39.5
17	f	11	n	y	2	2	y	2	2	1	y	y	y	75	nd
18	f	7	n	n	0	0	y	2	2	2	n	n	n	1002	30.3
19	m	14	n	y	2	1	y	2	2	2	n	n	n	608	54.2
20	m	13	y	y	1	2	y	2	2	1	y	n	n	214	57.3
21	m	13	y	n	0	0	y	2	2	2	n	n	nk	225	11
22	f	10	y	n	0	0	y	2	2	2	n	y	n	600	38.8
23	m	15	y	n	0	0	y	2	2	1	n	y	y	nd	5.95
24	m	12	y	n	0	0	y	2	2	2	y	n	nk	325	23.2
25	m	13	y	y	2	2	y	2	2	2	y	n	n	nd	>100
26	m	11	n	n	0	0	n	0	0	0	n	n	y	396	44.1
27	f	14	y	y	1	1	y	1	1	1	y	y	n	nd	nd
28	m	15	y	y	1	1	y	2	2	2	y	y	n	467	100
29	m	8	n	y	2	2	y	2	2	1	y	n	n	121	nd
30	m	15	y	n	0	0	y	1	1	1	y	n	n	656	45.4
31	m	8	y	y	2	2	y	2	1	1	y	y	y	674	253
32	m	13	y	n	0	0	y	2	2	1	n	y	n	846	>100
33	m	11	n	n	0	0	y	2	1	1	y	y	n	nd	47.5

34	m	10	y	n	0	0	y	2	1	1	y	n	n	nd	nd
35	m	6	n	n	0	0	y	2	2	1	n	n	n	221	class 3
36	m	9	y	y	1	1	y	1	2	1	y	y	n	78.3	1.06
37	m	8	y	y	1	1	y	2	2	1	y	y	nk	478	46.2
38	m	9	y	y	2	2	y	2	2	2	n	n	n	261	2.82
39	m		y	y	2	2	y	2	1	2	n	y	y	559	46.4
40	m	14	y	y	1	1	y	1	2	2	n	y	y	nd	41.9
41	m	12	y	y	1	1	y	1	1	2	y	n	n	121	1.54
42	m	11	y	n	0	0	y	2	2	2	y	n	n	85	33.5
43	m	8	y	y	2	2	y	2	1	2	n	y	n	1698	24.3
44	f	10	n	n	0	0	y	1	1	1	n	n	n	39.3	1.9
45	m	14	n	y	2	3	y	2	2	2	y	n	n	227	19.6
46	m	10	n	n	0	0	y	2	2	2	y	y	n	1557	90.2
47	m	11	y	y	1	2	y	2	1	1	y	y	n	804	99.6
48	m	9	y	y	2	2	y	2	2	2	y	y	y	954	57.8
49	m	11	y	n	0	0	y	2	2	1	y	y	n	1867	>100
50	m	11	y	n	0	0	y	2	2	1	y	n	n	491	>100
51	m	13	n	y	1	2	y	2	2	1	y	n	n	1140	>100
52	m	12	y	n	0	0	n	0	0	0	y	y	y	1440	67.1
53	m	12	y	y	0	0	y	2	1	2	y	y	y	694	>100
54	m	17	n	n	0	0	y	2	2	1	n	n	n	678	1.48
55	m	15	n	n	0	0	y	1	1	2	y	n	y	249	1.59
56	m	12	y	y	2	nk	y	1	2	2	y	y	y	1180	1.1
57	f	10	y	n	0	0	y	2	2	2	n	y	n	417	2.12
58	m	8	y	y	2	1	y	2	1	1	n	y	n	360	1.05
59	m	9	n	n	0	0	y	2	1	2	n	n	n	nd	1.53
60	m	6	y	y	3	1	y	1	2	2	n	y	n	105	nd
61	m	16	y	n	0	0	y	1	1		n	n	y	99.5	nd
62	m	10	n	y	2	1	y	1	2	1	n	y	nk	nd	2.32
63	m	11	n	n	0	0	y	2	1	2	n	n	nk	336	nd
64	m	14	y	n	0	0	y	2	2	2	y	n	n	439	13.5

f, female; m, male; y, yes; n, no; nd, not done; nk, not known; AD, atopic dermatitis; g6, ImmunoCAP to timothy grass pollen (Thermo Fisher, Uppsala, Sweden);
Asthma severity grading (GINA guidelines): 0, no; 1, intermittent; 2, mild persistent; 3, moderate persistent; 4, severe;
Asthma phenotype: 0, no; 1, post-viral; 2, atopic; 3, exercise-induced; 4, other;
Rhinitis severity grading (ARIA guidelines): 0, no; 1, mild; 2, moderate;
Rhinitis persistence: 0, no; 1, intermittent; 2, persistent; Rhinitis phenotype: 0, no; 1, sneezing; 2, obstructive;

Supplementary Table S2. Demographic, clinical, and serological characteristics of control patients (n=9).

control	sex	age	family history	asthma	severity	pheno-type	rhinitis	severity	persistance	pheno-type	conjunc-tivitis	AD	Food Allergy	total IgE kU/L	g6 kUA/L	SPT grass mix	SPT timothy grass	other sensitizations
1	f	17	n	y	1	2	y				n	n	n	323	0	0	0	mites
2	f	10	n	n	0	0	y	2	2	2	n	y	n	nd	0	0	0	mites
3	m	13	n	n	0	0	y	2	2	2	y	y	n	nd	nd	nd	0	alternaria
4	m	13	y	n	0	0	y	1	1	1	n	y	n	nd	nd	0	0	olive
5	m	8	y	n	0	0	y	1	1	1	n	y	n	86	0	0	0	olive
6	m	9	n	y	2	2	y	1	2	2	n	n	n	161	0	0	0	mites, molds
7	m	14	n	y	1	2	y	2	2	2	n	y	n	455	0	0	0	mites, molds, parietaria
8	m	11	n	n	0	0	y	1	2	2	n	y	n	339	0	0	0	mites
9	f	14	y	n	0	0	y	2	1	1	y	n	n	1285	0	0	0	olive

f, female; m, male; y, yes; n, no; nd, not done;

Supplementary Table S3. Wheal diameters (mm) are displayed for natural grass pollen extract, recombinant grass pollen allergens, the hybrid, and the histamine control.

patient	grass mix	timothy grass	histamine	Phl p 1	Phl p 2	Phl p 5	Phl p 6	Hybrid	histamine
1	10	6.5	7.5	4.5	0	3.5	0	5	4.5
2	5.5	0	6	3	0	0	0	4	3
3	4.5	6	7	4.5	6.5	5	3.5	4	5
4	6	5	4	4.5	0	10.5	4.5	3.5	4
5	7.5	4	6	4	0	7.5	11	4	4
6	4.5	4	20	3.5	2.5	0	0	3	9
7	6	4	6	4	7	4.5	2	4	3.5
8	3	3	5	4.5	0	4.5	5.5	3	3
9	4	3	4.5	5	0	0	0	3	4
10	7	4	7.5	5	7	4	0	4	4.5
11	0	3.5	nd	4	0	8	0	3	4
12	7	4.5	nd	4.5	0	6.5	0	4.5	4
13	5.5	4	3	2	8	5.5	3	3	2.5
14	5.5	4.5	6.5	3.5	0	5	0	3	4.5
15	4	8.5	7.5	3	8.5	7.5	7.5	5	3.5
16	8	7	3.5	5.5	6	4	5.5	4.5	4.5
17	4.5	5.5	5	3	0	0	0	3	4.5
18	3.5	3.5	5.5	3	0	4.5	0	4.5	5.5
19	6.5	3	5.5	5	4.5	0	13	6	5.5
20	6.5	7	6	4.5	0	9	4.5	6.5	6
21	4.5	3	5	0	0	3.5	0	3	5
22	6	8	5.5	4	0	9	5	9	5.5
23	7	4	6	0	0	4	0	3	6
24	8.5	6	5	3.5	6	5.5	2.5	4.5	4
25	6.5	5.5	4.5	3.5	5	7	7	4	nd
26	5.5	4.5	4	3	0	0	0	3	4
27	9	4.5	6	3.5	0	0	0	4	6
28	7.5	6	7	2.5	6.5	8	11.5	4.5	5
29	3.5	3	4.5	2	2	2	0	3	4.5
30	5.5	10	4.5	0	0	5.5	8.5	7	4.5
31	7.5	9	5	6	12.5	5	5.5	4	4
32	10.5	6.5	7	5	9	7.5	10	9	7
33	5.5	3	6	2.5	6	10.5	7	8	6
34	6	6	5.5	3	0	7	0	4.5	5.5
35	6	4.5	5.5	3	4.5	5.5	0	3	5.5
36	4.5	4.5	5	0	0	7.5	4	4	6
37	17	5	4.5	3.5	4	0	0	3	4.5
38	6	6	6.5	4	0	0	0	3.5	5
39	4	3	7	3	0	0	0	3	nd
40	6	4	3.5	3.5	0	0	0	3	nd
41	5	4	4	0	0	8	0	3	4
42	9	10	4.5	4	0	11	7	5	6
43	7.5	7.5	6	4	4	4.5	0	5	4.5
44	5.5	5	5	3.5	0	4	0	5	5.5
45	7.5	7	6	4	0	0	0	4	8.5

46	7.5	3.5	3.5	0	0	5	3.5	3	6
47	9	5	6.5	3	0	8	0	3	5
48	5	4	11	0	0	7.5	3.5	4	6
49	5.5	11.5	7	5	4	8.5	12	12	5
50	8	4	6.5	2.5	5	8.5	4	4	6.5
51	15	7.5	7.5	4	6.5	5	8	6	6
52	6	11.5	4.5	3.5	15	13	9	5	6
53	8	5	4	3	8.5	6	12	5	4.5
54	0	0	8,5	0	0	0	0	0	5
55	5.5	3.5	5	0	0	2	2	2	3.5
56	3	0	5	0	0	0	0	0	7
57	7	3	6.5	0	0	0	0	0	4.5
58	4	3	5	0	0	0	0	0	4
59	6	2	4.5	0	0	2	0	0	4.5
60	3	2	5	0	0	0	0	0	0
61	5	3	5.55	3.5	0	0	0	0	nd
62	4.5	2.5	7	2	0	0	0	0	7
63	2.5	2.5	5.5	1.5	0	0	0	0	4
64	3.5	3	4.5	0	0	0	0	0	5.5

nd, not done; wheal diameters ≥ 3.0 are considered positive (grey).

Supplementary Table S4. Phl p 1-, Phl p 2-, Phl p 5-, and Phl p 6-specific IgE levels (ISU) as determined by allergen microarray are displayed for patients 1-64. Grey fields indicate a positive SPT result to the respective allergen.

patient	Phl p 1	Phl p 2	Phl p 5	Phl p 6
1	0.98	0	0	0
2	32.06	0.37	17.02	1.07
3	24.7	3.77	54.31	5.48
4	12.21	0	55.64	0.92
5	6.1	0	53.9	4.84
6	0.48	0	0	0
7	4.75	1.94	20.68	0.63
8	4.97	0	52.33	22.79
9	0.41	0	0	0
10	50.97	1.8	1.39	0.25
11	0.6	0	1.19	0
12	0.23	0.12	1.31	0
13	13.51	1.02	11.31	0.25
14	3.1	0	5.5	0
15	63.19	8.12	47.85	11.08
16	21.06	1.48	9.8	3.5
17	0.64	0	0	0
18	5.85	0	4.65	0.3
19	37.36	2.3	0	0
20	0.14	0	11.11	0.9
21	0	0	3.68	0
22	26.15	0	14.84	0.51
23	0	0	1.2	0
24	8.7	0.27	8.79	0.14
25	20.06	1.52	38.76	0.4
26	9.32	0	0.19	0
27	1.38	0	0	0
28	12.18	1.94	45.65	2.77
29	0.3	0	0.22	0
30	4.22	0	25.38	3.61
31	38.64	2.6	23.08	2.16
32	16.9	32.05	20.92	3.66
33	9.5	1.69	16.97	5.63
34	35.71	0	23.79	0.29
35	18.59	0.36	1.23	0.1
36	0.62	0	4.71	0.27
37	29.54	1.15	0	0
38	6.92	0	0	0
39	1.15	0	0	0
40	9.43	0.13	0	0
41	0	0	4.75	0
42	9.9	0	10.32	0.8
43	1.67	1.5	1.63	0.2
44	1.85	0	0.94	0

45	15.44	0	1.24	0
46	102.2	0	21.36	5.17
47	61.64	0	45.69	1.01
48	0.87	0	15.22	0.74
49	83.57	0.17	25.95	51.95
50	65	2.39	111.51	11.16
51	34.67	49.21	63.98	13.31
52	21.59	16.13	1.05	0.36
53	37.51	15.43	41.89	14.29
54	0	0	0	0
55	0.51	0	0	0
56	1.26	0	0	0
57	4.92	0	0	0
58	0	0	0	0
59	0.56	0	0	0
60	0	0	0	0
61	7.14	0	0	0
62	7.36	0	0	0
63	45.12	0	0	0
64	0.32	0	0	0

Cut off ≥0.3 ISU

Supplementary Table S5. Number of patients (n=64) with positive (ISU+) or negative (ISU-) IgE levels and positive (SPT+) or negative (SPT-) skin reaction to Phl p 1, Phl p 2, Phl p 5, and Phl p 6.

	**Phl p 1**	**Phl p 2**	**Phl p 5**	**Phl p 6**
ISU+/SPT+	41	19	39	22
ISU-/SPT-	6	42	22	35
ISU+/SPT-	16	1	2	4
ISU-/SPT+	1	2	1	3

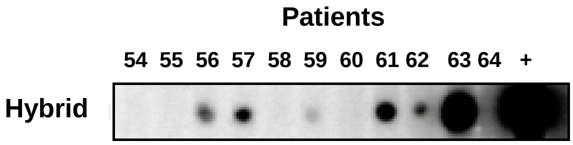

Figure S1. IgE-reactivity to the dotted hybrid of 11 patients without skin reactivity to the hybrid, and of a control serum from a grass pollen allergic patient (+). Bound IgE was detected with an iodine^{125}labelled anti-IgE antibody.

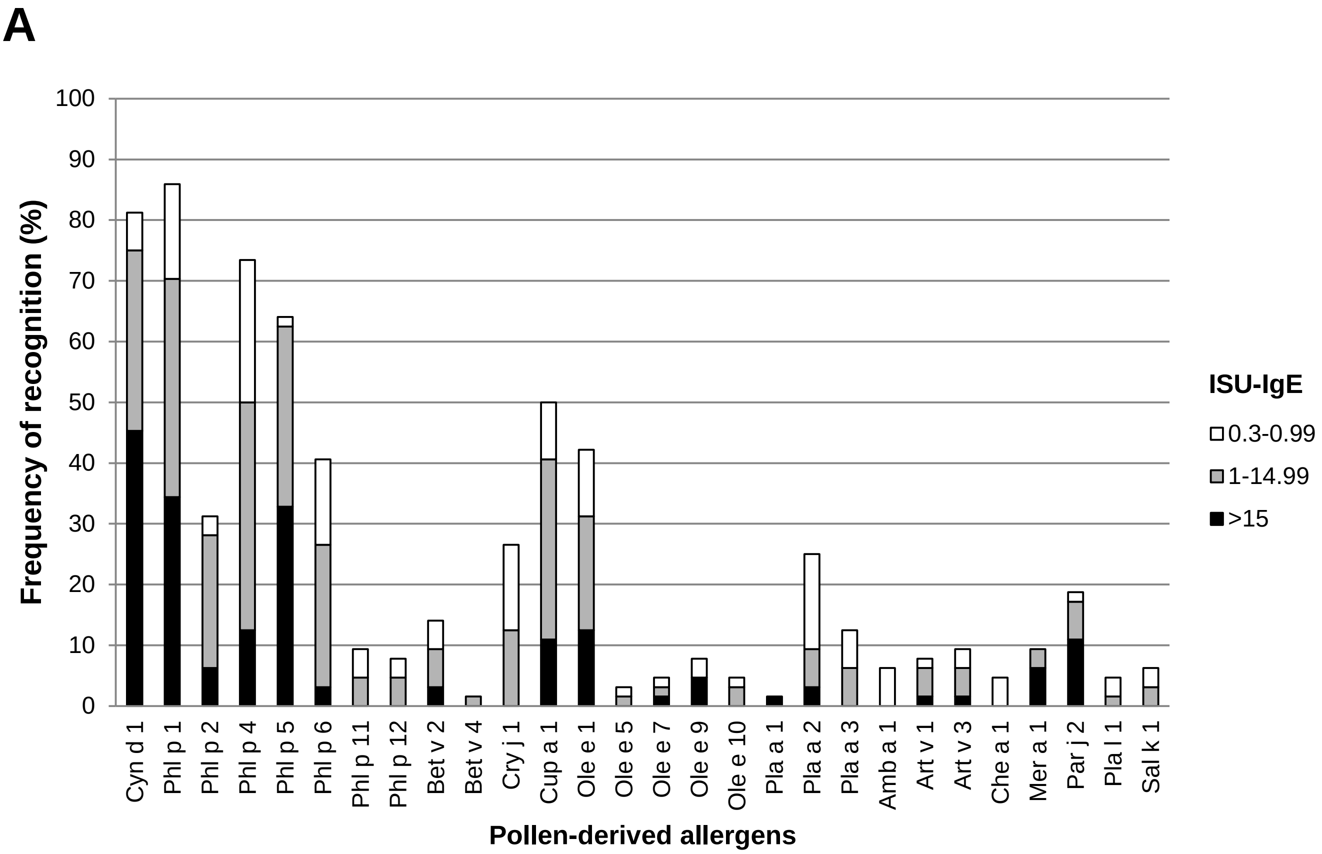
A
Frequency of recognition (%)
0
10
20
30
40
50
60
70
80
90
100
Cyn d 1
Phl p 1
Phl p 2
Phl p 4
Phl p 5
Phl p 6
Phl p 11
Phl p 12
Bet v 2
Bet v 4
Cry j 1
Cup a 1
Ole e 1
Ole e 5
Ole e 7
Ole e 9
Ole e 10
Pla a 1
Pla a 2
Pla a 3
Amb a 1
Art v 1
Art v 3
Che a 1
Mer a 1
Par j 2
Pla l 1
Sal k 1
Pollen-derived allergens
ISU-IgE
0.3-0.99
1-14.99
>15

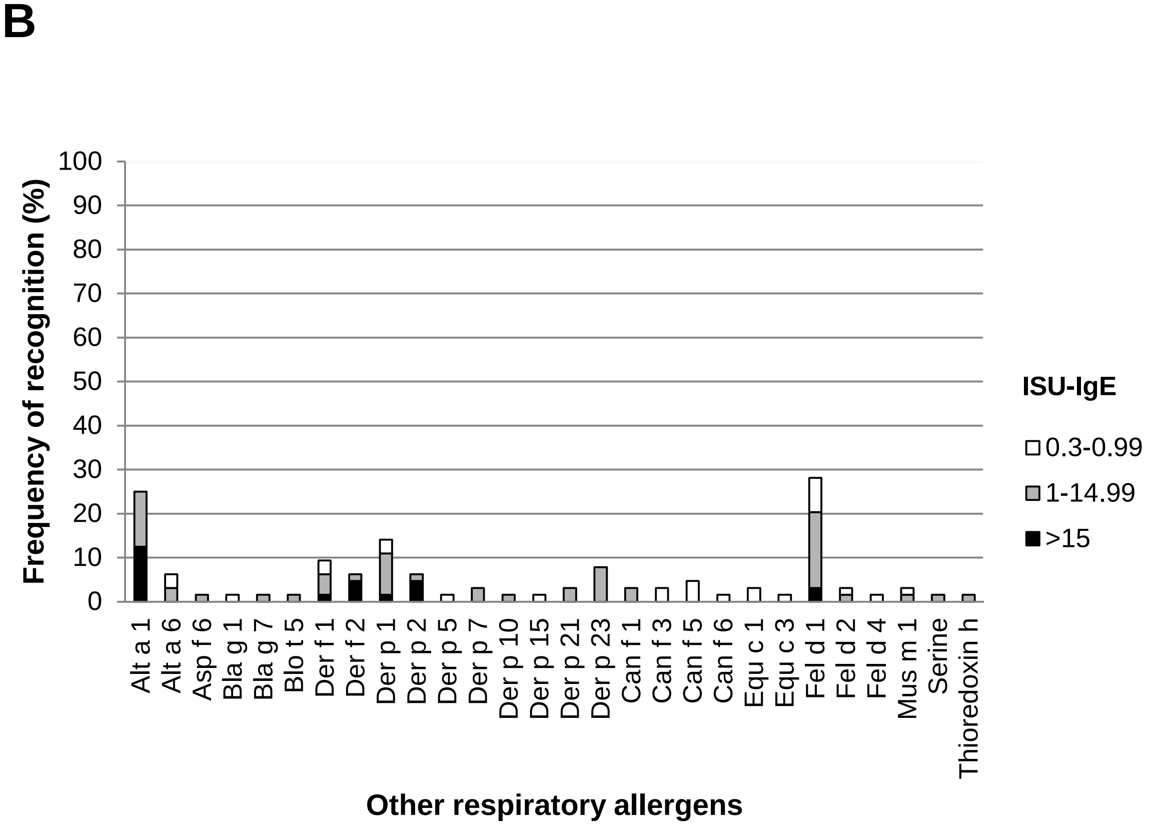
B
Frequency of recognition (%)
0
10
20
30
40
50
60
70
80
90
100
Alt a 1
Alt a 6
Asp f 6
Bla g 1
Bla g 7
Blo t 5
Der f 1
Der f 2
Der p 1
Der p 2
Der p 5
Der p 7
Der p 10
Der p 15
Der p 21
Der p 23
Can f 1
Can f 3
Can f 5
Can f 6
Equ c 1
Equ c 3
Fel d 1
Fel d 2
Fel d 4
Mus m 1
Serine
Thioredoxin h
Other respiratory allergens
ISU-IgE
0.3-0.99
1-14.99
>15

Figure S2

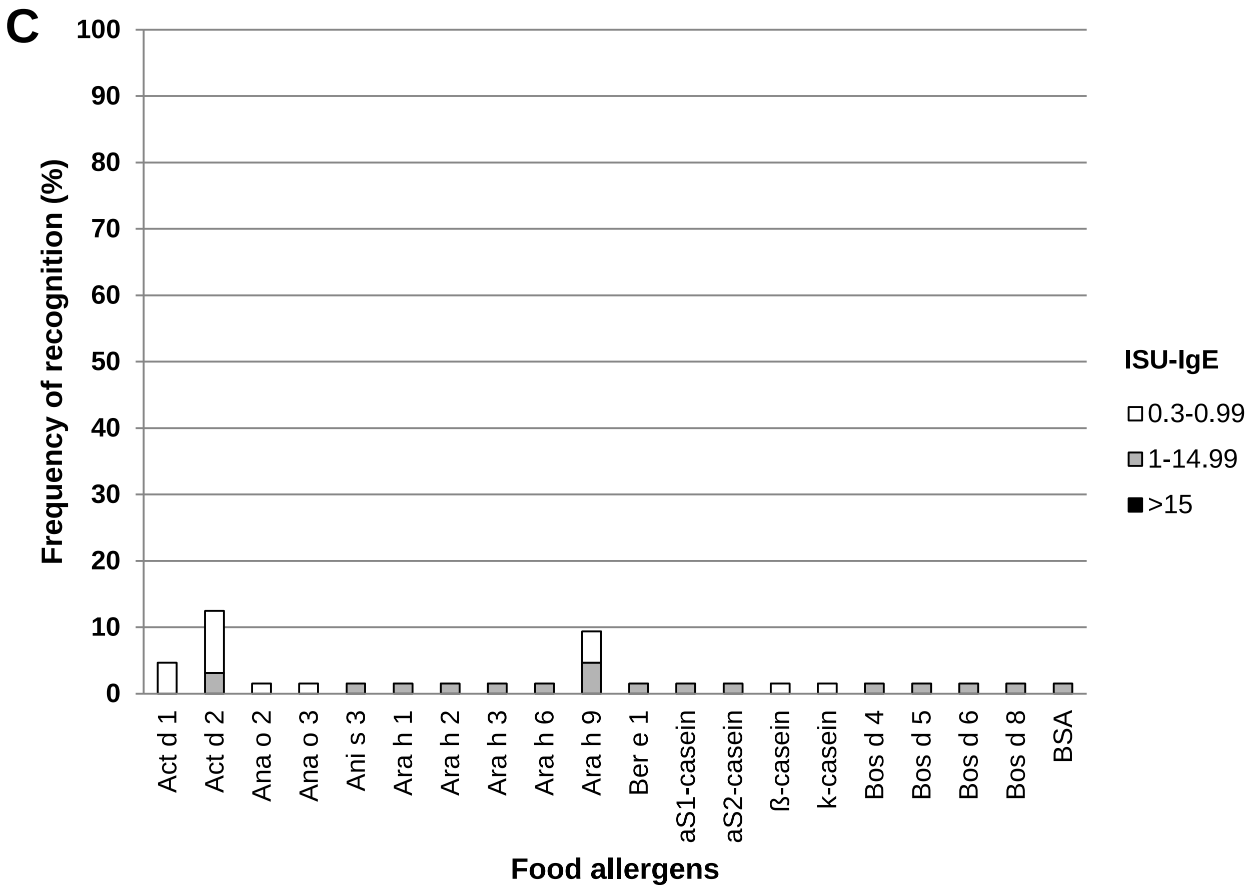
C
100
90
80
70
60
50
40
30
20
10
0
Frequency of recognition (%)
ISU-IgE
0.3-0.99
1-14.99
>15
Act d 1
Act d 2
Ana o 2
Ana o 3
Ani s 3
Ara h 1
Ara h 2
Ara h 3
Ara h 6
Ara h 9
Ber e 1
aS1-casein
aS2-casein
ß-casein
k-casein
Bos d 4
Bos d 5
Bos d 6
Bos d 8
BSA
Food allergens

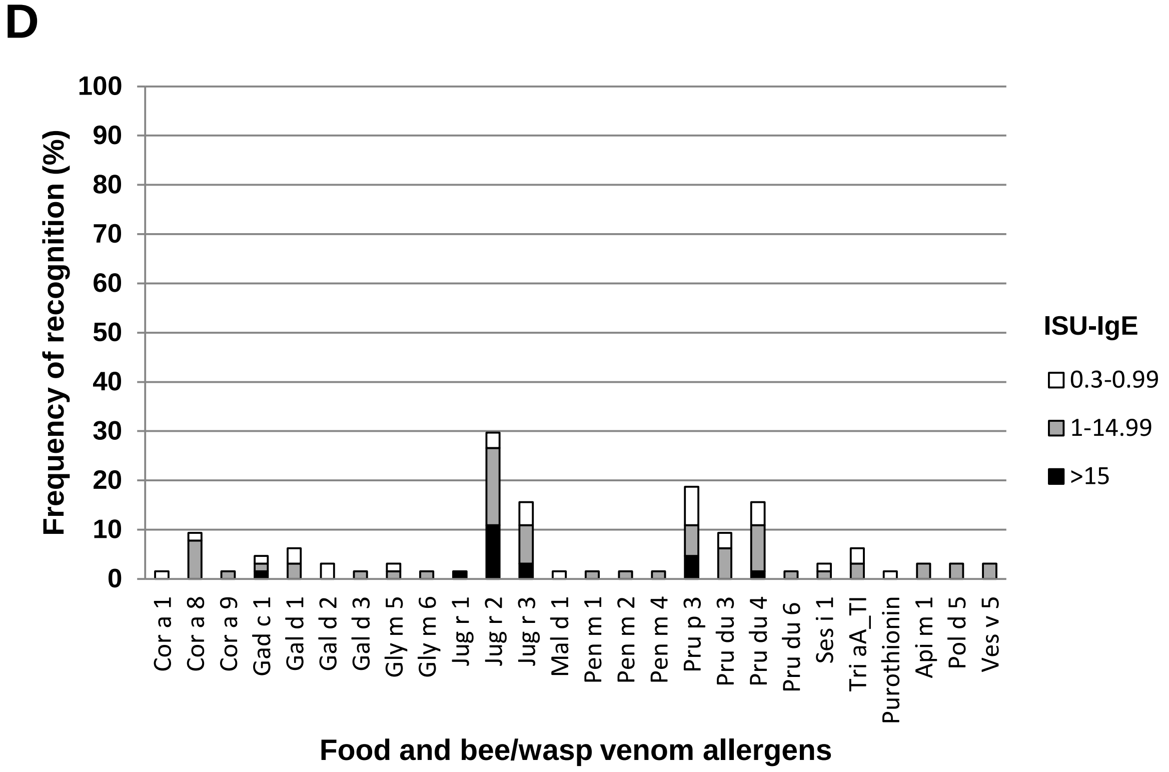
D
100
90
80
70
60
50
40
30
20
10
0
Frequency of recognition (%)
ISU-IgE
0.3-0.99
1-14.99
>15
Cor a 1
Cor a 8
Cor a 9
Gad c 1
Gal d 1
Gal d 2
Gal d 3
Gly m 5
Gly m 6
Jug r 1
Jug r 2
Jug r 3
Mal d 1
Pen m 1
Pen m 2
Pen m 4
Pru p 3
Pru du 3
Pru du 4
Pru du 6
Ses i 1
Tri aA_TI
Purothionin
Api m 1
Pol d 5
Ves v 5
Food and bee/wasp venom allergens

E

List of allergens on the MeDALL chip with negative test results

Allergen	Allergen-source	Origin	Name/Function of the protein/Allergen family
Alpha Gal	*Red meat*	R	*Gal-α1-3Gal-β1-3GlcNAc epitope*
Act d 5	*Kiwi*	N	Kiwellin
Act d 8	*Kiwi*	R	PR-10
Aln g 1	*Alder*	R	PR-10
Amb a 4	*Ragweed*	R	Defensin-like protein
Amb a 5	*Ragweed*	R	Polcalcin (2 EF-hand calcium binding protein)
Amb a 6	*Ragweed*	R	LTP
Amb a 9	*Ragweed*	R	Polcalcin (2 EF-hand calcium binding protein)
Amb a 10	*Ragweed*	R	Polcalcin (3 EF-hand calcium binding protein)
V Ana o 1	*Cashew nut*	N	Vicilin-like protein
Ani s 1	*Anisakis*	R	Serine protease inhibitor
Ani s 3	*Anisakis*	R	Tropomyosin
Api g 1	*Celery*	R	PR-10
Api m 1	*Bee venom*	R	Phospholipase A2
Api m 2	*Bee venom*	R	Hyaluronidase
Api m 4	*Bee venom*	R (peptide)	Melittin
Ara h 1	*Peanut*	R	Storage protein, 7S globulin
Ara h 8	*Peanut*	R	PR-10
Asp f 1	*Aspergillus*	R	Mitogillin family
Asp f 3	*Aspergillus*	R	Peroxysomal protein
Bet v 1	*Birch*	R	PR-10
Bla g 2	*Cockroach*	R	Aspartic protease
Bla g 5	*Cockroach*	R	Glutathione S-transferase
Bos d Lf	*Cow's Milk*	N	Transferrin
Can f 2	*Dog*	R	Lipocalin
Can f 4	*Dog*	R	Lipocalin
Cla h 8	*Cladosporium*	R	Mannitol dehydrogenase
Cor a 14	*Hazelnut*	R	Storage protein, 2S albumin
Der p 4	*House dust mite*	R	Alpha-Amylase
Der p 11	*House dust mite*	R	Paramyosin
Der p 14	*House dust mite*	R	large lipid transfer protein
Der p 18	*House dust mite*	R	chitinase like protein
clone 16	*House dust mite*	R	Chitin binding protein
Lep d 2	*Storage mite*	R	Mites, group 2
Fag e 2	*Buckwheat*	N	Storage protein, 2S albumin
Gal d 5	*Egg yolk/chicken*	N	Livetin (Serum Albumin but species specific)
Gly m 4	*Soy*	R	PR-10

Hev b 1	*Latex*	R	Rubber elongation factor
Hev b 3	*Latex*	R	Small rubber particle protein
Hev b 5	*Latex*	R	Acidic protein
Hev b 6.01	*Latex*	R	Hevein
MUXF3	*CCD*	N	CCD
rOle e 6	*Olive*	R	Acidic protein which mediates olive allergy
rOle e 8	*Olive*	R	Calcium-binding protein
Phl p 7	*Timothy grass*	R	Polcalcin
Pis v 3	*Pistachio*	R	7S Vicilin-like Globulin
Pru p 1	*Peach*	R	PR-10
Transferrin	*Cow's Milk*	N	Transferrin
Tri a 14	*Wheat*	R	LTP
Tri a 19.0101	*Wheat*	N	Omega 5 gliadin
Tri a 191_369	*Wheat*	R	Fragment of LMW Glutenin
Tri a 36	*Wheat*	R	Glutenin Subunit, LMW Glutenin
m43	*m43*	R	Fragment of HMW Glutenin Bx7
m82	*m82*	R	Fragment of HMW Glutenin Bx7
38/GTT	*#38/Glutathione transferase*	R	Glutathione transferase
112/1-Cys	*#112/1-Cys-peroxiredoxin*	R	Peroxiredoxins (1-Cys-peroxiredoxin)
126/Dehy	*#126/Dehydrin*	R	Dehydrins
Ves v 1	*Wasp*	R	Phospholipase
Beta amylase	*Wheat*	R	beta-Amylase
Avenin	*Wheat*	R	Avenin
GG1	*Wheat*	R	Gamma-gliadin
0MYO	*Human*	R	Neg. control for 2N Myoglobin
2MYO	*Human*	R	CCD-Marker

Figure S2. IgE sensitization profile of grass pollen allergic children (n=64) as determined by the MeDALL allergen-chip. The frequency of recognition (%) (y-axis) of selected (A) pollen-derived allergens, (B) other respiratory allergens, and (C) food allergens, and (D) food and bee/wasp venom allergens (x-axis) are displayed. ISU levels measured for each allergen were grouped (white bars: 0.3-0.99 ISU, grey bars: 1-14.99 ISU, black bars: ≥15 ISU). (E) List of allergens on the MeDALL chip with negative test results.
